# Hospital Reform

**Published:** 1891-12-26

**Authors:** George Henty

**Affiliations:** 302, Camden Road, N.


### Editor's Letter-Box.

[Our correspondents are reminded that prolixity is a great bar to publication, and that brevity of style and conciseness of statement greatly facilitate early insertion.]

### HOSPITAL REFORM.

Sir,—In an article in a late number of your valuable journal you highly commended the executive of the Great Northern Central Hospital, Holloway-road, for their adoption of the system of careful inspection and registration of out-patients, and causing an inquiry into their condition of life, so that the charity, being a free hospital, should not be imposed upon, nor the interests of the private medical practitioners made to suffer from so much readiness to give medical advice and medicine in such institutions. I agree with you that great credit is due to the managers of that new hospital for commencing this system of preventing so much fraud, by allowing persons to obtain so easily medical assistance, when many of them are well able to remunerate the hard-working medical men in the neighbourhood of hospitals.

There is, too, another great evil which will require much agitation and discussion, with your help, before we can alter or prevent, and that is the belief among the working classes that they have a right to eleemosynary medical relief at the various hospitals. No doubt that idea is founded on and perpetuated by the plan so generally adopted of giving governors' letters or orders in proportion to their subscriptions, without any discrimination of the position or means of the patient to whom such letter or order is given, and the sooner we can disabuse the public mind of the falsity of this plan the better. It has been said that a *noble* governor has declared it to be his right to send his servants or dependents to a hospital for all or any of its benefits. Is this charity? Shall the bequests for the poor be swallowed up by such practices? Shall Governors who are *not* noble, but able, to help the charity continue to take from it, rather than leave the benefits to the absolute poor, for it is only for them that hospitals were first intended and endowed, and for whose benefit they ought still to be exclusively confined?

Your efforts will be much required in keeping this great question before the public, and especially the hospital Governors, for it has been much discussed for many years by medical men and medical associations. In the year 1886 the British Medical Association appointed a deputation to wait on the Hospitals Association to discuss the best means of enforcing this question on all hospital authorities. The Hospitals Association, under the presidency of Sir Andrew Clark, did discuss the matter very earnestly, and Dr. Dickson and myself proposed, in the name of the Council of the British Medical Association, "That in the opinion of this Council, all hospitals should adopt the plan of an enquiry into the circumstances of all out-patients, it being entrusted to a paid officer of the hospital; and that Governors should cease to grant out-patient letters, unless to persons whom, from their personal knowledge they know to be deserving, and in a condition to require hospital relief." This matter remains in *statu quo*; nothing has been done either by the Hospitals Association or any other body. Some say there is no authority unless the State interferes, and great hopes are entertained that the Lords' Committee on Medical Charities will report in favour of such a view; and as we know they have heard much evidence in support of these principles, we trust they will order or suggest the same plan to be carried out. This conviction of opinion it is necessary to force on the public mind by agitation and discussion in medical journals, and especially on the minds of the rising generation of working men, who, rather than depend on thrift and careful living to raise their condition, think that if they contribute one penny a week to the Saturday Fund they may claim as a right "gratuitous advice and medicine" at all the London and country hospitals. For many years the managers of the London Hospital, to their great credit, have adopted the system, and now we have the youngest hospital doing the same. I should hope the nine or ten others will be persuaded in time to adopt this effective plan, and that it may be equitably carried out throughout this great metropolis.—Yours faithfully,

George Henty, M.D.

302, Camden Road, N., December, 1891.

